# Marker selection strategies for circulating tumor DNA guided by phylogenetic inference

**DOI:** 10.1093/bioinformatics/btaf145

**Published:** 2025-03-31

**Authors:** Xuecong Fu, Zhicheng Luo, Yueqian Deng, William LaFramboise, David Bartlett, Russell Schwartz

**Affiliations:** Department of Biological Sciences, Carnegie Mellon University, Pittsburgh, PA 15217, United States; Department of Biological Sciences, Carnegie Mellon University, Pittsburgh, PA 15217, United States; Ray and Stephanie Lane Computational Biology Department, Carnegie Mellon University, Pittsburgh, PA 15217, United States; Allegheny Health Network Cancer Institute, Allegheny Health Network, Pittsburgh, PA 15212, United States; Allegheny Health Network Cancer Institute, Allegheny Health Network, Pittsburgh, PA 15212, United States; Department of Biological Sciences, Carnegie Mellon University, Pittsburgh, PA 15217, United States; Ray and Stephanie Lane Computational Biology Department, Carnegie Mellon University, Pittsburgh, PA 15217, United States

## Abstract

**Motivation:**

Blood-based profiling of tumor DNA (“liquid biopsy”) offers great prospects for non-invasive early cancer diagnosis and clinical guidance, but requires further computational advances to become a robust quantitative assay of tumor clonal evolution. We propose new methods to better characterize tumor clonal dynamics from circulating tumor DNA (ctDNA), through application to two specific tasks: (i) applying longitudinal ctDNA data to refine phylogeny models of clonal evolution, and (ii) quantifying changes in clonal frequencies that may be indicative of treatment response or tumor progression. We pose these through a probabilistic framework for optimally identifying markers and using them to characterize clonal evolution.

**Results:**

We first estimate a density over clonal tree models using bootstrap samples over pre-treatment tissue-based sequence data. We then refine these models over successive longitudinal samples. We use the resulting framework for modeling and refining tree densities to pose a set of optimization problems for selecting ctDNA markers to maximize measures of utility for reducing uncertainty in phylogeny models and quantifying clonal frequencies given the models. We tested our methods on synthetic data and showed them to be effective at refining tree densities and inferring clonal frequencies. Application to real tumor data further demonstrated the methods’ effectiveness in refining a lineage model and assessing its clonal frequencies. The work shows the power of computational methods to improve marker selection, clonal lineage reconstruction, and clonal dynamics profiling for more precise and quantitative assays of somatic evolution and tumor progression.

**Availability and implementation:**

https://github.com/CMUSchwartzLab/Mase-phi.git. (DOI: 10.5281/zenodo.14776163).

## 1 Introduction

The discovery of circulating free DNA (cfDNA) in human blood and the observation that tumor-derived cfDNA may occur at greatly elevated levels compared to DNA of healthy cells—e.g. due to elevated release of tumor cell DNA, abnormal clearance of DNA debris from cell death, or circulating tumor cells in blood—established the potential for liquid biopsy, i.e. blood-based profiling of solid tumor genomics (Crowley *et al.* 2013, Wan *et al.* 2017). Rapid, non-invasive profiling of tumor states offers many possibilities for improving cancer diagnosis and treatment (Cescon *et al.* 2020), including early prognosis (Phallen *et al.* 2017, Connal *et al.* 2023) and detecting residual disease and relapse (Mattox *et al.* 2019, Ignatiadis *et al.* 2021). Liquid biopsy methods have now been studied widely in various cancer types (Maia *et al.* 2020, Kemper *et al.* 2023). The technology has its limitations, however, mainly due to the challenge of separating tumor signals from the influence of much larger numbers of healthy cells and the consequent need for highly sensitive genomic assays. Deep sequencing on liquid biopsy samples with low signal-to-noise ratio is one option but can be too costly and slow for repeated use. Alternative molecular testing methods have thus been used, including multiplex-PCR (Abbosh *et al.* 2017), droplet digital PCR (ddPCR) (Huerta *et al.* 2021), and strategies for enriching for tumor DNA with targeted sequencing (Phallen *et al.* 2017, Kurtz *et al.* 2021). These technologies offer a path to highly sensitive quantitation of somatic variants found at low levels in the blood, although with the tradeoff of profiling relatively few pre-selected markers.

Despite its broad potential, current clinical application of liquid biopsy has primarily been for prognosis or recurrence detection (Reinert *et al.* 2019, Sanz-Garcia *et al.* 2022), rather than more precise quantitative analysis of tumor genetics. While there is now a rich literature on characterizing tumor evolutionary trajectories from numerous forms of genomic assays (c.f., Beerenwinkel *et al.* 2015, Schwartz and Schäffer 2017), the need to work typically with low precision or relatively limited blood-based marker sets makes it infeasible to incorporate longitudinal blood samples in a straightforward way into current methods for tumor phylogenetics. Yet there is also little work to date on developing new classes of inference method suitable for effectively bringing liquid biopsy into tumor phylogeny models. One notable exception has been recent work from the TRACERx Consortium using a tumor-specific phylogenetic method to profile ctDNA from non-small-cell lung cancer patients (Abbosh *et al.* 2017). That study inferred a base phylogenetic tree for each patient with primary multi-regional sequencing and then used PCR on liquid biopsy samples, preoperative and post-operative, to track clonal and subclonal populations. The same team later investigated a larger cohort with metastasis using a new PCR technique and bioinformatics tool tailored for ctDNA to track the clonal lineages longitudinally (Abbosh *et al.* 2023). Their work showed that liquid biopsy samples can identify marker mutations of distinct subclones and characterize clonal population changes in metastases or relapses, provided they can draw on an accurate model of clonal lineages. However, much remains unaddressed with regard to how to identify optimal markers for use in such analyses and how to use these most effectively to determine the clonal lineage model and how its population frequencies evolve over time. While problems of experimental design for follow-up studies in tumor phylogenetics have been considered previously (Weber *et al.* 2020), probe selection for liquid biopsy presents special challenges that have not to our knowledge been previously considered.

This work is aimed at developing methods to better characterize clonal dynamics of tumors from liquid biopsy data. We focus on challenges that have not, to our knowledge, been addressed in prior work. First, we examine the question of how we can leverage ctDNA data using small marker sets to refine phylogenetic models so as to correct errors, reduce uncertainty in inference, or expand a tree to accommodate variants or clones not seen in earlier samples. Second, we consider the question of optimal marker selection: for typical scenarios in which one must select a small subset of markers to profile with high sensitivity, which markers are likely to be most informative? We examine this question for selecting markers to optimally refine the tumor phylogeny model and to optimally characterize changes in clonal frequency or tumor heterogeneity over time. We then show on simulated and real data that our methods allow one to apply liquid biopsy so as to accurately capture dynamics of clonal population changes in tumors, with potential application to various tasks in tumor diagnostics, monitoring, and clinical decision-making.

## 2 Materials and methods

**Figure 1.**
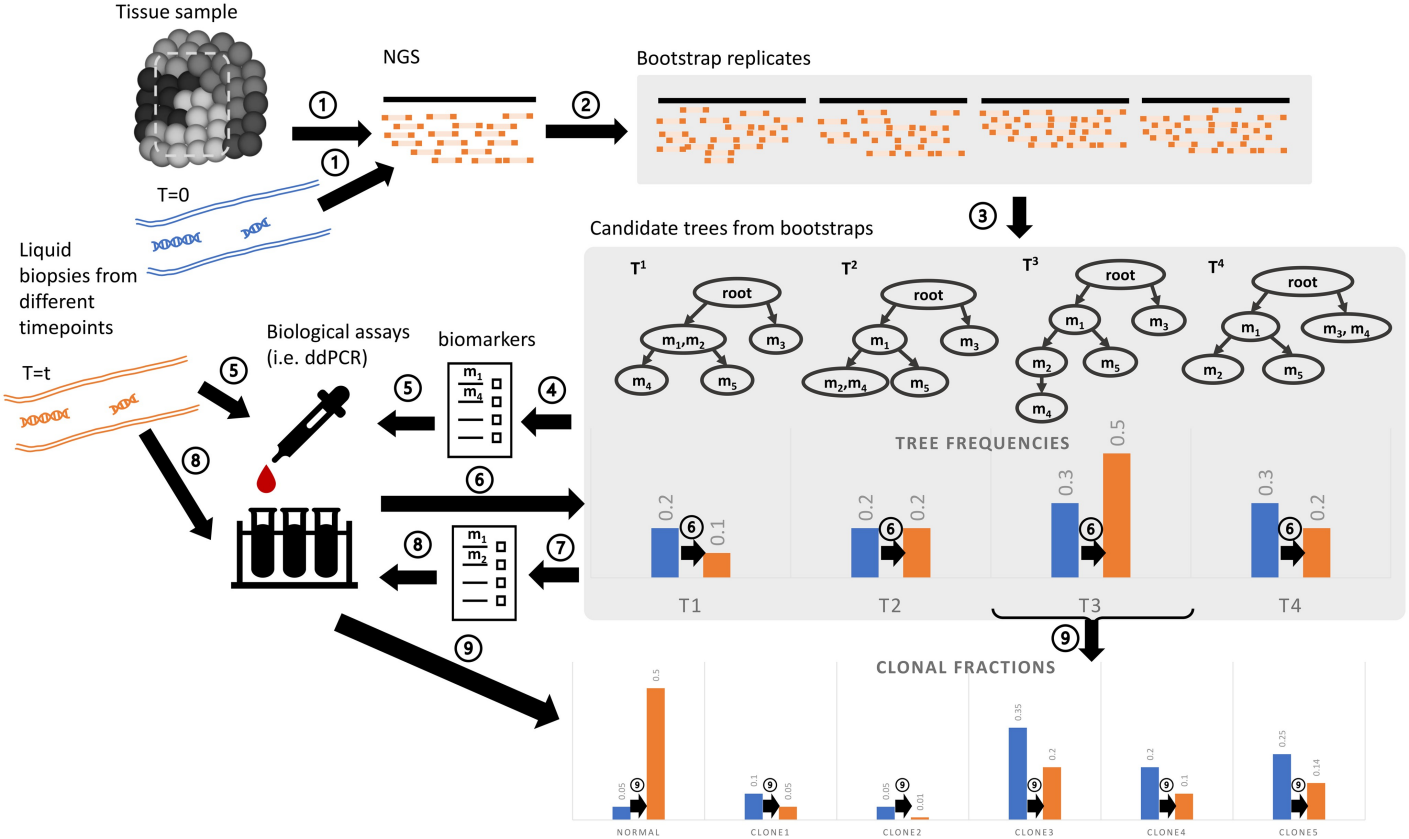
Our overall inference pipeline. (1) We assume we have first sequenced tissue and liquid biopsy sample(s), obtaining reference germline and ctDNA sequence. (2) We create bootstrapped samples over reads for each sequence set. (3) We infer a set of possible trees from the bootstrapped samples, establishing an estimated empirical tree density. (4) We then seek a set of optimal biomarkers of mutations to best reduce the tree uncertainty and (5) quantify these markers in ctDNA (e.g. by ddPCR). (6) We use the results of these assays to update the empirical tree densities. (7) We further seek a set of optimal biomarkers to track subclone frequencies efficiently and (8) assay these biomarkers. (9) Finally, we use the results of the assays to estimate clonal fractions at each sampled time point.

In this section, we consider variants of the problem of marker selection for liquid biopsy with the goal of developing personalized assays that allow rapid longitudinal corrections on a patient-specific basis. For each variant, we assume we need to select a small marker set for high-precision assays, such as by ddPCR. For simplicity of exposition, we refer to these high-precision assays as ddPCR, but another more quantitatively precise assay, such as high-depth targeted sequencing, might also be used. We solve the problem for the general case of assuming that we might select any observed mutation as a marker for any given subject. However, the problem is conceptually the same if we are limited to choosing a subset of markers from a larger predefined set, e.g. selecting from a set of markers for which PCR probes are already available. We first consider the problem of choosing markers to refine a phylogenetic model and reduce uncertainty in clonal lineage inference. We then consider selection with the goal of characterizing changes in clonal frequencies for a given tree or density of tree models. The overall workflow for simultaneously addressing these questions is shown in Fig. 1.

### 2.1 Problem 1: Selecting mutation markers to minimize uncertainty in phylogenetic inference

We first consider the problem of choosing liquid biopsy markers to reduce uncertainty in tree inference. Due to the low signal-to-noise of ctDNA, we expect ctDNA genomic measurements to yield poor results with standard phylogenetic inference tools. We provide evidence for this assertion in Supplementary Results Section S2.1 and Supplementary Fig. S2. As a result, we assume high uncertainty in tree inference and so pose phylogenetic inference in terms of densities of trees rather than a single optimum. For the present work, we estimate this density through bootstrapping over sequence reads, an approach that allows us to use existing tumor phylogeny methods that are designed to return a single optimal tree. Bootstrapping over reads effectively gives us a sample over possible VAFs consistent with the data, which can in turn manifest in a density over tree topologies, mutation placements on the trees, and clonal frequencies. Bayesian phylogeny methods might provide a more principled alternative to capture the initial tree distribution, although designing an efficient Bayesian sampler for non-trivial tumor phylogeny models is a challenging problem in itself. Once we have an initial tree density, we then seek in part to select liquid biopsy markers that will allow us to reduce uncertainty in the tree density by facilitating comparisons that provide evidence for or against some topologies, by finding marker sets that will best distinguish between possible high-confidence models in the initial tree density.

#### 2.1.1 Problem formulation

We first establish a general probabilistic framework for defining a tree density and posing optimal marker selection problems over it. In subsequent sections, we adapt this framework to some problem variants, each of which involves estimating a density over trees and optimizing marker selection for a desired objective over that density. The notation below is a formalization of this idea that can be adapted to different problem assumptions and objectives.

Given a candidate (bootstrapped) tree set $T={\left\{T^{k}\right\}}_{k=1,\ldots ,K}$ we define a clonal tree structure matrix $E^{k}$ that specifies possible trees for a set of defined tumor clones. For each tree k, $E_{i,j}^{k}=1$ if clone (tree node) i is the parent of node j and otherwise 0. We define a mutation assignment matrix $M^{k}$ where $M_{i,l}^{k}=1$ if mutation $g_{l}$ belongs to node i for each tree k and otherwise 0. We define a clonal frequency array $F^{k}={\left(F_{i}^{k}\right)}_{i=1,\ldots ,N}$, encoding the latent variant allele frequency (VAF) of each of N observed mutations in each clone i for each tree k. We further define a set of N gene markers (mutations) $\left\{g_{1}\ldots g_{N}\right\}$. We want to select n≤N gene markers by finding a mapping f:[n]→[N] identifying the markers such that $E_{T^{q}\in T}E_{T^{k}\in T\setminus \{T^{q}\}}\;\text{log}\;E_{(R^{f},S^{f})∼T^{k}}P(R^{f},S^{f}|T^{q})$ is minimized, where $R^{f}=(r_{1},r_{2}\ldots r_{n})$ is a family of random variables describing ddPCR read counts for a set of probes and $S^{f}$ is a binary matrix mapping biomarkers to clones in the tree structure for an underlying ground truth tree $T^{k}$. In the model, we assume that the generation of read counts is i.i.d. and independent from the generation of the tree structures.

We split the likelihood of observing a given dataset into the probability of observing the read counts and the probability of observing the tree structures. Assume that the read count for each gene marker $g_{f(j)}$ is $r_{j}$. Then letting $M^{k}(l)=i$ when $M_{i,l}^{k}=1$:

(1) $$\begin{array}{c} E_{(R^{f},S^{f})∼T^{k}}P(R^{f},S^{f}|T^{q})\\ =E_{(R^{f},S^{f})∼T^{k}}P(R^{f}|F^{q},M^{q})P(S^{f}|E^{q})\\ =E_{R^{f}∼T^{k}}P(R|F^{q},M^{q})E_{S^{f}∼T^{k}}P(S|E^{q})\\ E_{R^{f}∼T^{k}}P(R|F^{q},M^{q})=\sum_{r_{j}}\prod_{j=1}^{n}P(r_{j}|F_{M^{q}(f(j))}^{q})=① \end{array}$$

Since the markers we are selecting are intended to be used for a high-precision assay, canonically ddPCR but potentially some form of high-depth sequencing, we assume that the probability of observing the read counts is close to a normal distribution for computational convenience. Assume the read depth is D. Then assuming $r_{j}∼N(\mu_{j}^{k},{\left(\sigma_{j}^{k}\right)}^{2})$, $\mu_{j}^{k}=DF_{M^{k}(f(j))}^{k}$, ${\left(\sigma_{j}^{k}\right)}^{2}=DF_{M^{k}(f(j))}^{k}(1-F_{M^{k}(f(j))}^{k})$:

$$P(r_{j}|F_{M^{q}(f(j))}^{q})=\frac{1}{\sqrt{2\pi {\left(\sigma_{j}^{q}\right)}^{2}}}\text{exp}\;\left(-\frac{{\left(r_{j}-\mu_{j}^{q}\right)}^{2}}{2{\left(\sigma_{j}^{q}\right)}^{2}}\right)$$

where $\mu_{j}^{q}=DF_{M^{q}(f(j))}^{q},{\left(\sigma_{j}^{q}\right)}^{2}=DF_{M^{q}(f(j))}^{q}(1-F_{M^{q}(f(j))}^{q})$.

Then:

$$\begin{array}{c} ①=\int_{r_{1},\ldots ,r_{n}}\prod_{j=1}^{n}P(r_{j}|F_{M^{q}(f(j))}^{q})d(r_{1},\ldots ,r_{n})\\ =\prod_{j=1}^{n}\frac{1}{\sqrt{2\pi ({\left(\sigma_{j}^{q}\right)}^{2}+{\left(\sigma_{j}^{k}\right)}^{2})}}\;\text{exp}\;\left(-\frac{{\left(\mu_{j}^{k}-\mu_{j}^{q}\right)}^{2}}{2({\left(\sigma_{j}^{q}\right)}^{2}+{\left(\sigma_{j}^{k}\right)}^{2})}\right)\\ \;\text{log}(①)=-\frac{1}{2}\sum_{j=1}^{n}\;\text{log}\;(2\pi ({\left(\sigma_{j}^{q}\right)}^{2}+{\left(\sigma_{j}^{k}\right)}^{2}))-\frac{1}{2}\sum_{j=1}^{n}\frac{{\left(\mu_{j}^{k}-\mu_{j}^{q}\right)}^{2}}{{\left(\sigma_{j}^{q}\right)}^{2}+{\left(\sigma_{j}^{k}\right)}^{2}} \end{array}$$

For probabilistic modeling of tree structure perturbations, we use the ancestor-descendant distance (Govek *et al.* 2020) to specify a distance between subtrees. We define a subtree $S_{f}^{k}$ of $T^{k}$ given the mapping f defining a set of n marker genes using their ancestor-descendent matrix A. For $T^{k}$, $A_{i,j}^{k}=1,i,j=1,\ldots N$ if mutation i is the ancestor of j. Then for $S_{f}^{k}$, ${\hat{A}}^{k}{\left(f\right)}_{i,j}=1$ if $A_{f(i),f(j)}^{k}=1,i,j=1,\ldots ,n$. For each unit change of AD distance, we assign a regularization factor λ≥1 defining a likelihood function on markers. Therefore, up to a constant factor

(2) $$\begin{array}{c} E_{S^{f}∼T^{k}}P(S^{f}|E^{q})=E_{S^{f}∼T^{k}}P({\hat{A}}^{k}(f)|{\hat{A}}^{q}(f))\\ \;\;\;\;\;\propto \lambda^{\left|{\hat{A}}^{k}(f)-{\hat{A}}^{q}(f)\right|} \end{array}$$

 

$$\begin{array}{c} \text{log}\;E_{S^{f}∼T^{k}}P(S^{f}|E^{q}(f))=\sum_{i=1}^{n}\sum_{j=1}^{n}\left(\left|{\hat{A}}_{i,j}^{k}(f)-{\hat{A}}_{i,j}^{q}(f)\right|\right)\;\text{log}\;\lambda +C \end{array}$$

for an arbitrary constant C that we can ignore in the optimization. Combining the two components yields an objective function that we seek to minimize in marker selection:

(3) $$\begin{array}{c} \underset{f}{\min}E_{T^{q}\in T}E_{T^{k}\in T\setminus \{T^{q}\}}\;\text{log}\;E_{(R^{f},S^{f})∼T^{k}}P(R^{f},S^{f}|T^{q})\\ =-\sum_{q=1}^{K}\sum_{k=1,k\neq q}^{K}(-\frac{1}{2}\sum_{j=1}^{n}\;\text{log}\;(2\pi ({\left(\sigma_{j}^{q}\right)}^{2}+{\left(\sigma_{j}^{k}\right)}^{2}))\\ -\frac{1}{2}\sum_{j=1}^{n}\frac{{\left(\mu_{j}^{k}-\mu_{j}^{q}\right)}^{2}}{{\left(\sigma_{j}^{q}\right)}^{2}+{\left(\sigma_{j}^{k}\right)}^{2}}+\sum_{i=0}^{n}\sum_{j=0}^{n}\left(\left|{\hat{A}}_{i,j}^{k}(f)-{\hat{A}}_{i,j}^{q}(f)\right|\right)\;\text{log}\;\lambda ) \end{array}$$

This objective function provides a way of evaluating the utility of a given marker set for reducing a measure of uncertainty in expectation over the tree density and sampling of sequence reads. We use this objective to perform marker selection for the purpose of optimal tree refinement by integer linear programming (ILP) over possible choices of markers and over trees in the tree density to find a marker set optimizing for the objective function (3) in expectation, as described in more detail below.

### 2.2 Problem 2: Refining tumor phylogenetic tree distribution using ctDNA assays

For the ddPCR counts tested for selected marker genes $g_{1}$ and $g_{2}$, suppose without loss of generality that $g_{1}$ is the ancestor of $g_{2}$ in a proposed tree structure. We then refine the tree structure by testing which trees are plausible given the set of relationships among $g_{1}$ and $g_{2}$ consistent with their read counts. While one might build this into the tree likelihood and solve *de novo* for the tree density, for efficiency reasons we instead apply new marker data to refine an existing density over trees. We assume in the discussion below that the read depths for each tree are described by binomial variables based on the allele frequencies and overall read depth, i.e. $R_{i}^{j}∼\text{Bin}(\mathrm{dept}h_{j},f_{g_{i}})$ describes a read count of gene marker i in tree j, for which tree j has a total read count $\mathrm{dept}h_{j}$ and the gene marker i has allele frequency $f_{g_{i}}$. Below, we continue to use S to represent the tree structure, $R^{0}$ for the observed read counts from the original primary tissue sequencing, and $R^{1}$ for the observed PCR counts from the liquid biopsy samples.

We use a Bayesian approach to capture changes in our inferred tree density by updating the weights for each possible tree topology to reflect its plausibility given all of the data seen to date. Since we used bootstrap trees to approximate the initial tree density, we define the initial weight of each tree structure to be the count of that tree structure observed in the bootstrapping. These counts are normalized to produce a probability density over trees, but are represented initially as integers here. Then:

(4) $$\begin{array}{c} P(S|R^{1},R^{0})\propto P(S,R^{1}|R^{0})=P(R^{1}|S,R^{0})P(S|R^{0})\\ =P(R^{1}|S)P(S|R^{0})=\int_{f}g(R^{1},f|S)P(S|R^{0}) \end{array}$$

where $P(S|R^{1},R^{0})$ defines the updated weights, $P(S|R^{0})$ is the original weights, f describes the possible VAFs for the mutation markers from liquid biopsy, and $g(R^{1},f|S)$ is the probability density of a particular set of clonal VAFs f and the corresponding read counts given a tree structure S.

We develop the special case of two markers to derive the basic method for refining the tree topology and for use in subsequent illustration. We generalize the method to multiple independent marker pairs through sequential application of pairwise updates. A more rigorous but tractable generalization to k markers for arbitrary k is less trivial and left as an exercise for future work. For two markers, there are four types of relationship those markers might take on in a tree structure: (a) marker 1 is an ancestor of marker 2, meaning that $f_{1}\geq f_{2}$. (b) marker 2 is an ancestor of marker 1, meaning $f_{1}\leq f_{2}$, (c) marker 1 and marker 2 belong to the same clone, meaning $f_{1}=f_{2}=f$, (d) marker 1 and marker 2 belong to different branches of the tree, meaning $f_{1}+f_{2}\leq 1$. Therefore, let $R_{1}^{1},R_{2}^{1}$ be the read counts of marker 1 and 2 from liquid biopsy and $D_{1}^{1},D_{2}^{1}$ be the read depths. Then:

$$\int_{f_{1},f_{2}}g(R_{1}^{1},R_{2}^{1},f_{1},f_{2}|S)=\left(\begin{array}{c} D_{1}^{1}\\ R_{1}^{1} \end{array}\right)\left(\begin{array}{c} D_{2}^{1}\\ R_{2}^{1} \end{array}\right)\times$$

 

$$\{\begin{array}{cc} \int_{0}^{1}\int_{0}^{f_{1}}f_{1}^{R_{1}^{1}}{\left(1-f_{1}\right)}^{D_{1}^{1}-R_{1}^{1}}f_{2}^{R_{2}^{1}}{\left(1-f_{2}\right)}^{D_{2}^{1}-R_{2}^{1}}df_{1}df_{2}, & \mathrm{case}(a)\\ \int_{0}^{1}\int_{f_{1}}^{1}f_{1}^{R_{1}^{1}}{\left(1-f_{1}\right)}^{D_{1}^{1}-R_{1}^{1}}f_{2}^{R_{2}^{1}}{\left(1-f_{2}\right)}^{D_{2}^{1}-R_{2}^{1}}df_{1}df_{2}, & \mathrm{case}(b)\\ \int_{0}^{1}f^{R_{1}^{1}}{\left(1-f\right)}^{D_{1}^{1}-R_{1}^{1}}f^{R_{2}^{1}}{\left(1-f\right)}^{D_{2}^{1}-R_{2}^{1}}df, & \mathrm{case}(c)\\ \int_{0}^{1}\int_{0}^{1-f_{1}}f_{1}^{R_{1}^{1}}{\left(1-f_{1}\right)}^{D_{1}^{1}-R_{1}^{1}}f_{2}^{R_{2}^{1}}{\left(1-f_{2}\right)}^{D_{2}^{1}-R_{2}^{1}}df_{1}df_{2}, & \mathrm{case}(d) \end{array}$$

Equation (4) provides a formula to update the weight of each possible tree by multiplying each weight by the above integral. We then normalize the overall updated weights over all observed tree structures to sum to 1 in order to yield an updated tree density. In Supplementary Methods S1.1, we also describe a more conservative alternative to the Bayesian update method, in which we pose tree density updates via statistical hypothesis testing to reject a subset of an existing tree density that is inconsistent with new ctDNA marker frequencies.

### 2.3 Problem 3: Selecting markers to track subclonal populations

We next consider another criterion for marker selection: effectively tracking changes in subclonal population frequencies. We would typically assume that our purpose in conducting liquid biopsy is to track changes in clonal frequencies or derived measures of overall heterogeneity that might be indicative, e.g. of recurrence after treatment, expansion of a resistant clone, or metastasis. Intuitively, to optimize for this criterion, we want to avoid choosing redundant markers that mark the same clone but rather find a set of markers that are distributed across the phylogeny so as to provide as much power as possible to monitor changes in frequencies of distinct clones.

#### 2.3.1 Optimizing for a known tree

To better explain the approach, we first derive a solution for this problem under the assumption that we have determined a specific lineage tree with certainty. We later describe how to generalize that model to the actual case where we assume a density over a set of trees rather than a single known tree.

**Problem Statement:** Given the same input as in Section 2.1, and a most likely tree $\hat{k}$, find a mapping f:[N]→[n] identifying n gene markers such that the sum of weighted clones tracked by the markers in T is maximized.

For the present work, we set the weights to be the estimated clonal fractions from the previous time point, posing the problem so as to maximize the estimated fraction of the tumor tracked in the next sample by assuming frequencies are close to those at the prior time point. However, the weights here could be any arbitrary design. For example, weights might alternatively be designed to bias the solution to favor particular probes based on their expected clinical utility, ease of probe design, preference for probes already available, or some other prior knowledge.

For computational convenience, we define the weight $F_{i}^{k}$ for clone i of bootstrap tree k to be the weight of any mutations first appearing in clone i for tree k. Since a mutation originating at a node would be expected to be carried by the entire subtree rooted at that node, its weight would be the sum of frequencies of clones over that subtree. We use this observation to create an array of estimated clonal frequencies ${\bar{F}}_{i}^{k}=F_{i}^{k}-\sum_{j}F_{j}^{k}$ for i∈{i=1,…,Clone_num}, j∈Children(i). In normal use in longitudinal sampling, these weights would then update with each longitudinal time point to provide a best guess as to the weights at the next time point.

We then define a binary output vector z of length N to identify the chosen markers, where element j of z will be one if marker j is tracked and zero if it is not. Then we can define $x=\bar{M^{\hat{k}}z}$ to be an array where $x_{j}=1$ indicates the node j is tracked by marker set z and $x_{j}=0$ if not. $x^{T}\bar{F}$ is then the total proportion of the tracked clones for which we maximize.

Estimating clonal frequencies from marker VAFs is not entirely straightforward, though, because the mutations acquired in any node will be inherited by its descendent nodes. Therefore, identifying a single clone’s frequency requires measuring a marker of that clone and markers of its child clones. We call this the “complete information assumption” and the previously illustrated scenario, in which we effectively know the frequency of the entire subtree rooted at a clone, the “partial information assumption.” Under the complete information assumption, we create a matrix ${\hat{E}}^{\hat{k}}=E^{\hat{k}}+I$ mapping clones to mutations whose VAFs would allow us to identify the clonal fraction. We define a pairwise product ⊙ between matrix A and array b such that ${\left(A⊙b\right)}_{ij}=a_{ij}*b_{j}$. We define a row-wise sum of a matrix A as σ(A) where $\sigma {\left(A\right)}_{i}=\sum_{j}a_{ij}$. We can then pose the problem of finding the optimal marker set to correspond to solving the following constrained optimization problem relative to the optimal tree ${\hat{E}}^{\hat{k}}$: $\max_{z}\;t^{T}{\bar{F}}^{\hat{k}}$ such that $t=\text{abs}(1-\sigma ({\hat{E}}^{\hat{k}}⊙x^{\hat{k}}-{\hat{E}}^{\hat{k}}))$.

#### 2.3.2 Optimizing over a tree density

We next extend the simplified model, which assumes a known tree, to consider uncertainty in tree inferences by assuming we have a density over either a subset of candidate trees or the full tree space. Assume that $T^{{}^{\prime }}={\left\{T^{\bar{k}}\right\}}_{\bar{k}\in S\subset \{1,\ldots ,K\}}\subset T$ is the tree set over which we want to optimize. Our objective function would then become the expectation over the tree density of the partial information objective, $\max_{z}\sum_{\bar{k}\in S}{\left(x^{\bar{k}}\right)}^{T}{\bar{F}}^{\bar{k}}$, where $x^{\bar{k}}=\bar{M^{\bar{k}}z}$ under the partial information assumption. Under the complete information assumption, the constrained optimization problem is transformed to $\max_{z}\;\sum_{\bar{k}}{\left(t^{\bar{k}}\right)}^{T}{\bar{F}}^{\bar{k}}$ such that $t^{\bar{k}}=\text{abs}(1-\sigma ({\hat{E}}^{\bar{k}}⊙x^{\bar{k}}-{\hat{E}}^{\bar{k}}))$, similar to the deterministic tree case. To better clarify the problem, we provide a simple example of marker selection in Supplementary Methods S1.2 and the accompanying Supplementary Fig. S1.

### 2.4 Quantifying tumor subclonal populations

After refining the tumor phylogenetic tree distribution and choosing markers for subclonal population tracking, we next seek to quantify the subclonal populations using the chosen markers. We infer the frequency of a clone using the mean VAF of measured markers appearing in the given clone minus the sum of mean VAFs of markers of its child clones. Note that this model assumes that we only choose markers in copy number neutral regions and thus can treat VAF as a proxy for cancer cell fraction (CCF).

### 2.5 Implementation of the optimization methods

All probe optimization problems described here are solved by formulating them as integer linear programs (ILPs). We create a variable $z\in {\left\{0,1\right\}}^{N\times 1}$ where $z_{i}=1$ if ith mutation is selected as a marker and $z_{i}=0$ otherwise. We constrain $\sum_{i=1}^{N}z_{i}=n$ to fix a number n of markers to be used. We then optimize over z for each objective function, establishing an ILP. We use Python with Gurobi for the solver. We tested our problem with two tumor phylogeny methods, the MCMC-based method PhyloWGS (Deshwar *et al.* 2015) and a simplified version of our own ILP method, TUSV-ext (Fu *et al.* 2022), restricted to only consider SNVs. ILPs were all solved using Gurobi V.9 in the present work.

## 3 Results

### 3.1 Simulated data

We initially tested our methods on simulated data to verify their effectiveness when ground truth trees and clonal densities are known. Details on the simulations methods are provided in the Supplementary Methods S1.3. Additional results on those data guiding some design choices in the later experiments but omitted here due to space limitations appear in Supplementary Results S2.1.

**Figure 2.**
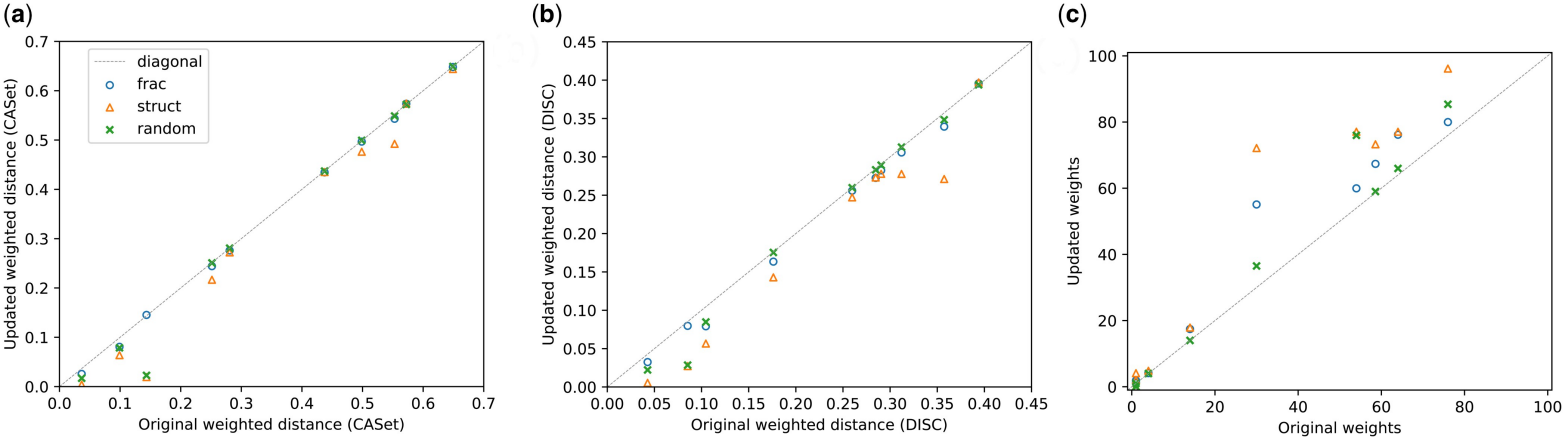
Results from 10 simulated clonal trees derived from 1 blood and 2 tissue samples, each with 7 clones, mutation rate 50, tissue mask proportion 0.5. (a) Updated weighted distance after tree adjustment versus original weighted distance between estimated and ground truth trees. CASet (Dinardo *et al.* 2020) is used as the distance metric. (b) The updated weighted distance after the tree adjustment versus the original weighted distance using DISC (Dinardo *et al.* 2020) as the metric. (c) Updated weights versus original weights for the best tree structure, with the lowest distance compared to the ground truth tree. We compared three marker selection strategies: optimizing for inferring clonal fractions (frac), inferring tree structures (struct), and random selection (random).

We tested the whole analysis pipeline of Fig. 1 on the simulated data. We first generated 100 bootstrapped samples for each simulation case and inferred a tree for each bootstrap replicate. Since PhyloWGS performed better in the earlier tests (Supplementary Section S2.1), we use it subsequently to generate bootstrap trees. We then apply our marker selection methods on the bootstrap tree sets before applying the chosen markers to adjust the tree distribution according to the simulated ddPCR data. Our simulation results show that using even limited numbers of markers can shift the tree distribution toward the ground truth tree; almost all cases yield refined trees below the diagonal in 2(a) and (b), meaning that the update reduces the weighted distance of the tree distribution to the ground truth tree. The weights of the bests tree relative to alternatives are also improved, as seen in Fig. 2c. We compare each of these measures for markers chosen for the purpose of reducing uncertainty, markers chosen for optimally characterizing clonal frequencies, and randomly selected markers. Markers chosen to optimize their ability to reduce uncertainty in the tree structure indeed perform substantially better at this task. Markers selected for their utility for estimating clonal frequencies perform more poorly at refining trees than those selected for the purpose of refining trees, as we would expect. Randomly chosen markers similarly perform poorly at updating tree topology. Random markers sometimes outperform markers chosen for inferring clonal frequencies at the task of refining trees and vice versa.

**Figure 3.**
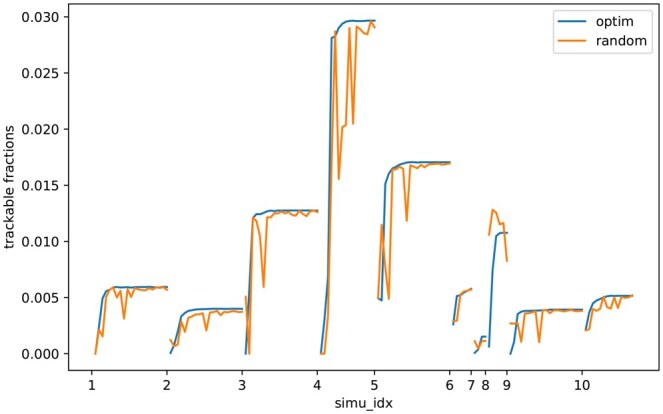
The trackable fractions of simulated tumor clonal population in noisy liquid biopsy samples. The lines show the change of trackable fractions, defined as the fraction of the total clonal frequency describing clones that can be quantified with the optional marker set, as the selected marker number increases. We compare marker selection optimized for the task of maximizing trackable fraction against random marker selection.

We also evaluated the ability of the methods to monitor clonal fractions. We visualize the results from ten simulation cases in Fig. 3, showing increasing ability to characterize clonal frequencies with larger numbers of optimally chosen markers. By comparison, inferences from randomly selected markers are typically unstable and require more markers to achieve minimal accuracy.

### 3.2 Application to a TRACERx lung cancer case

**Figure 4.**
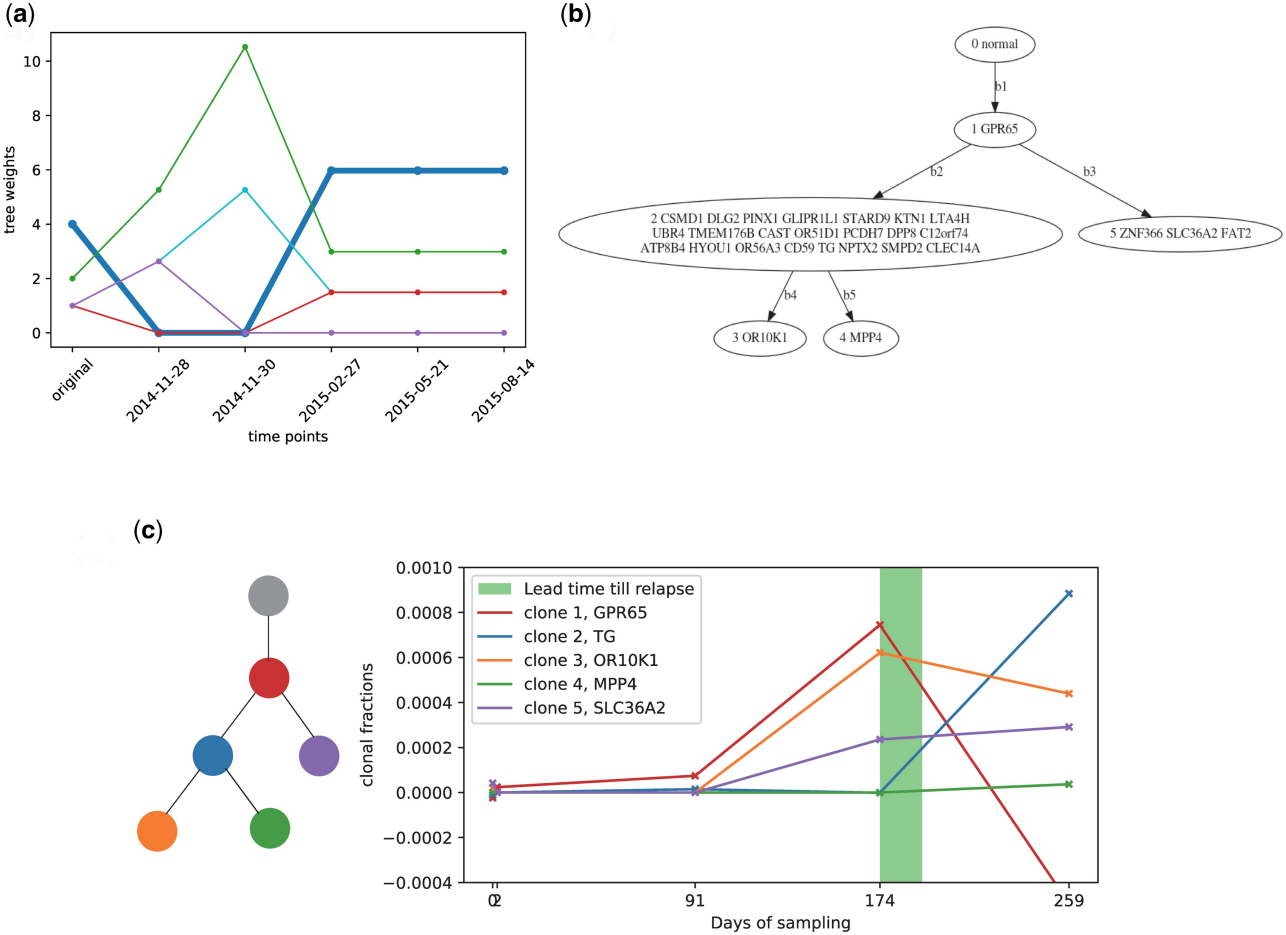
Results of applying our tree refinement and clonal tracking methods on TRACERx sample CRUK0044. (a) Changes in tree weights for each topology identified in bootstrap sampling, after adjusting the tree density using the selected markers at each time point. (b) The inferred most likely tree after all serial samples, corresponding to the blue line in (a). (c) Inferred clonal frequencies as of each longitudinal sample derived from the selected marker set, with lines representing the clones color-coded as in the tree at left.

Abbosh *et al.* (2017) assayed ctDNA for the first 100 TRACERx research participants, which Abbosh *et al.* (2023) expanded to additional participants. To demonstrate our methods, we selected one TRACERx case, CRUK0044, for which three primary tumor multi-regional samples were sequenced followed by six consecutive temporal liquid biopsy samples. Our simulation study suggested optimizing for tree structure provides a more informative basis for selecting markers than does optimizing for clonal frequencies, so we applied only tree structure based selection for this case. We iteratively selected a subset of markers for each time point from a larger marker set actually profiled by the TRACERx study, and used these to update the empirical tree densities (Fig. 4a). The blood samples reinforced the tree structure originally most frequently observed in bootstrap replicates from tissue sequence (thick blue line), suggesting that the initial multiregional sequencing had likely given highest weight to the correct tree. Nonetheless, the ctDNA allowed us to increase our confidence in that inference by providing evidence against other possible topologies that had been consistent with the original data. The first sample after the surgery tends to have the least ctDNA remaining, as we might expect, making the tree density adjustment less stable and temporarily leading to an inference that a lower-weight tree (green line in Fig. 4a) was the most plausible. However, the tree density stabilizes with subsequent blood draws with enhanced confidence in the initial most likely tree.

We then apply the second marker selection strategy to track the clonal subpopulations, using the most probable tree structure (Fig. 4b) as the presumed correct tree. Since we have three multi-regional samples, we have an initial candidate list of markers from all three samples. We select the top five markers among those called in at least two of the three tissue samples. We tracked the fraction of each subclonal population for each ctDNA time point (Fig. 4c). Our method is able to use just a small subset of markers to capture an overall trend of gradual and then accelerating expansion of several subclones after surgery indicative of relapse. This trend was apparent in the sampling by day 91, although several of the clones that came to dominate in relapse were seemingly present at negligible levels in the blood until day 174. We also note a sharp rise of the initially rare subclone 5, which then becomes the dominant clone by day 259 post relapse. We interpret this as likely indicating resistance of subclone 5 to the therapy applied after day 174, causing it to displace the initially common clones 1 and 3 as they respond to treatment. The data reveal some imprecision in inferences, however, most prominently in the inference of a negative frequency at day 259 for the once-dominant clone 1. A negative clonal frequency indicates that markers for the descendants of clone 1 are observed at higher frequency than markers for clone 1 itself, an impossible result if the tree and marker frequencies are exact (El-Kebir *et al.* 2015) but one that can occur due to imprecision in frequencies or error in the topology.

## 4 Discussion

We develop and apply a computational framework for interpreting ctDNA data for refining phylogenetic tree models and tracing clonal frequencies in tumor phylogenetic models. We use this framework to develop methods for optimally selecting limited numbers of high-sensitivity blood-based markers for tracking cancer progression. We pose and solve two variants of the marker selection problem: selecting markers to refine tumor phylogeny models and to track clonal frequencies. Application to simulated data shows the methods yield good accuracy at both tasks with limited numbers of markers, substantially improved over markers selected randomly or for a different task. Application to real data further demonstrates the potential of the methods to bring liquid biopsy more effectively to studies of clonal evolution and to clinical applications that depend on precisely and quantitatively tracking changes in clonal dynamics over time.

The present work is largely a proof of concept of a general approach to phylogeny-assisted marker selection for liquid biopsy, which might be extended in a number of ways. The modeling framework could be adapted to more sophisticated Bayesian tree models, e.g. to develop more principled but tractable strategies for larger marker sets. Other objective functions for defining optimal marker sets or interpreting their results might also be considered, tuned to specific clinical questions of interest. The general model might also be adapted to other ways of quantifying uncertainty and its minimization, e.g. by drawing on theory for minimizing over uncertainty developed in the context of active learning (c.f. Sverchkov and Craven 2017). We also note that the present application focused on SNVs, but in future work it will be important to consider structural variations (SVs), which are likely to be high impact and also make effective probes for tumor tracking, and copy number alterations (CNAs), which are often the mechanism of action of tumor driver genes and can confound interpretation of SNVs. Considerable work remains to take advantage of the new capabilities informatics can enable for liquid biopsy to lead to improvements in the practice of public health interventions and clinical treatment of cancers. Finally, we note that the strategies developed here might be adapted to other scenarios where one seeks to track clonal evolution of low-frequency cell populations obscured by a large neutral background, such as in tracking somatic evolution in non-cancerous tissues.

## Supplementary Material

btaf145_Supplementary_Data

## Data Availability

The data underlying this article are available in the article, in its online supplementary material, or with its accompanying code at https://github.com/CMUSchwartzLab/Mase-phi.git.
