# Supplementary material for: Marker selection strategies for circulating tumor DNA guided by phylogenetic inference
Source: Bioinformatics. 2025 Mar 31;41(4):btaf145. doi: 10.1093/bioinformatics/btaf145 (PMC12002908; doi:10.1093/bioinformatics/btaf145)
Supplement: btaf145_Supplementary_Data [file btaf145_supplementary_data.pdf]

Supplementary Material for Fu et al. – Marker selection strategies for circulating tumor DNA guided by phylogenetic inference

S1. Supplementary Methods

S1.1. Alternative significance testing method for refining tree density

An alternative to our Bayesian update approach for using ctDNA to refine a tree density is to apply statistical significance tests to accept or reject potential candidate trees based on whether the measured marker frequencies are plausibly consistent with a given tree topology. This significance test approach yields a stricter test than the Bayesian method, rejecting entire topologies but requiring stronger evidence to adjust the tree density than does the Bayesian update method. We assume here that frequencies of clones may change over time in ways that reveal some trees to be implausible that initially seemed plausible, but that the set of clones and their tree topology are unchanged over the course of the longitudinal sampling. Given a pair of markers, we can pose the question of whether their read counts at a specific point in time are consistent with a given tree topology as a statistical hypothesis test, here using a Wald test with the test statistic

$$W = \frac{\hat{\delta}}{\hat{se}} = \frac{\hat{f}_{g_2} - \hat{f}_{g_1}}{\sqrt{\frac{\hat{f}_{g_1}(1-\hat{f}_{g_1})}{depth_1} + \frac{\hat{f}_{g_2}(1-\hat{f}_{g_2})}{depth_2}}}$$

with size α. We reject the hypothesis when $W > z_\alpha$, with $\alpha = 0.05$ in the present work. For each pair of markers, we perform the Wald test with a Bonferroni-corrected α and then remove from the density all tree structures that are rejected by any pairwise marker test. Repeating this for all pairs of markers in a set then gives a general test to reject a portion of the tree density and lead to a refined density consistent with both the original sequence and subsequent ctDNA data. This test may thus be applied serially for multiple longitudinal assays, provided the original density was sufficiently well sampled that the correct tree is found within it.

S1.2. Illustrative example of marker selection for tracking clonal frequencies

To better explain the connection between marker selection and our ability to track clonal frequencies, we provide a simple illustrated example in Fig. S1. In this example, we consider a density of two trees of three clones each. We assume they are assayed on three markers and we wish to select two markers of those three that best characterize their clonal frequencies. In this case, the optimal solution is selecting markers m_2 and m_3, which allows one to track an expected 0.5 fraction of the total clonal density.

S1.3. Generating simulated tree densities

We create simulated phylogenetic trees parameterized by the number of subclones and the maximum degree of each subclone to control how many child subclones a parent node can have, randomizing subclone distributions to set up the total tree structure. We use a beta distribution to generate true allele frequencies for each subclone in this tumor tree. Based on the assumption that clones will have different frequencies at different tumor sites or in tissue versus blood, we used a Dirichlet probability distribution to randomize clonal frequencies independently in each tumor tissue. To mimic observations on real tumor and blood samples, we added a masking step so that only a part of the total subclones that are nearer the root are observed in tissue samples and the rest can only be observed in liquid biopsy samples. We normalize the fractions of observed clones in each tissue sample so that the fractions add up to one. However, in liquid biopsy tumor blood samples, normal cells may dilute the mutated alleles in the blood, which will lead to a higher frequency for the normal cell's alleles. Therefore, we add a frequency of 0.9 to the normal cell with a subsequent Dirichlet random variable to randomize the frequency for each subclone. We use an additional Poisson random variable to randomly assign a number of mutations to each subclonal node in the tree.

Since we already simulate the total structure of the tree, the assignment of mutations, and their corresponding variant allele frequencies, we are able to assign a depth of total reads for both tissue tumor samples and blood tumor samples. We then use a Poisson random variable to select for a total read count for each variant in each sample based on those numbers and use a binomial distribution with probability parameter equal to the variant allele frequency to sample a number of variant reads and reference reads.

To model sampled ddPCR measurements from this clonal growth model, we assign a number of droplets collected and set the number of these detected to be the mutant based on the normalized real data, using the mean of droplets in the given real sample and the known variant allele frequency to derive the number of droplets with the mutant allele.

The above protocol was applied to generate ten simulated instances each consisting of one blood and two tissue samples, seven clones, a mutation rate of 50, and tissue mask proportion 0.5. Mean depth of coverage was set to 800 for tissue samples to approximate a high depth targeted sequencing and to 3000 for blood sequencing meant to approximate ddPCR precision. Mutation numbers were stochastic and so varied by simulation. We did not perform any clustering of mutations.

For full implementation details needed to reproduce our analysis, readers may refer to the project Github:
https://github.com/CMUSchwartzLab/Mase-phi.git

S2. Supplementary Results

S2.1. Initial phylogeny construction

Before directly assessing our methods for marker selection and application, we first assessed how accurately we can determine phylogenies by different tumor phylogeny methods using simulated tissue and blood samples. Many tools have been developed for tumor phylogenetics

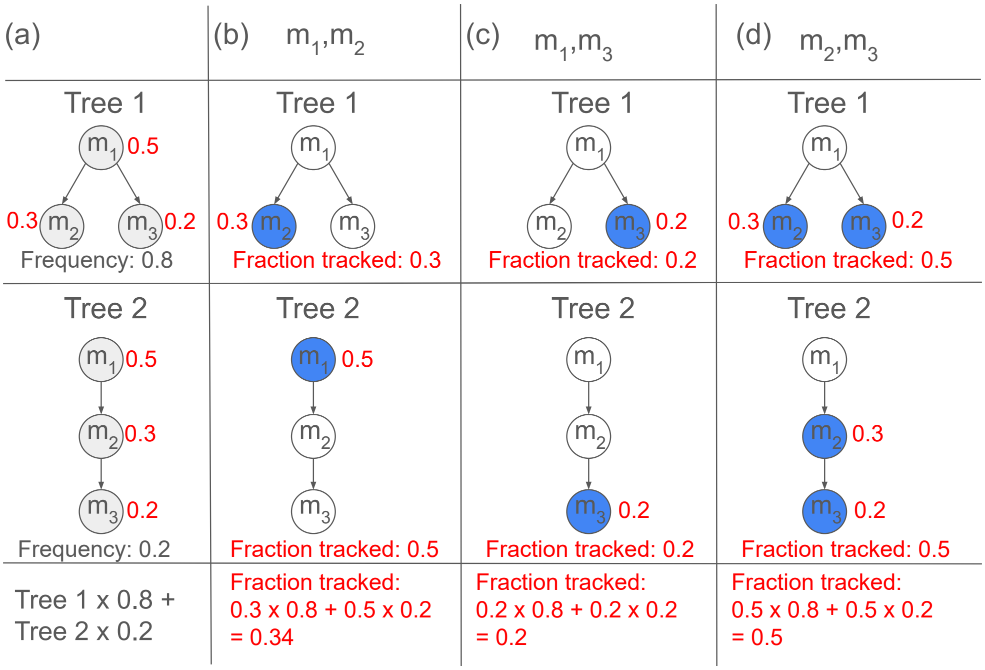

Fig. S1: Illustrative example of marker selection for tracking clonal densities in which we seek to select two markers from a set of three. (a) Illustration of a hypothetical tree density covering two trees, Tree 1 with probability 0.8 and Tree 2 with probability 0.2. Each tree is tracked by three markers — m_1, m_2, and m_3 — each of which is inferred to be first observed in a distinct clone. We assume in both trees there are three clones with estimated clonal frequencies of 0.5, 0.3, and 0.2. (b) Tracking in both trees if marker set $\{m_1, m_2\}$ is selected. For Tree 1, only the clone marked by m_2 is tracked since it is a leaf whose unique marker m_2 would provide its frequency. For Tree 2, the clone marked by m_1 is tracked since it can be estimated by $m_2 - m_1$. This yields an expected fraction 0.34 of total clonal density tracked across the tree density. (c) Tracking in both trees if marker set $\{m_1, m_3\}$ is selected. For Tree 1, only the clone marked by m_3 is tracked since it is a leaf whose unique marker m_3 would provide its frequency. For Tree 2, only the clone marked by m_3 is tracked since it is again a leaf node for which the prevalence of m_3 would provide an estimate of clonal frequency. This yields an expected fraction 0.2 of total clonal density tracked across the tree density. (d) Tracking in both trees if marker set $\{m_2, m_3\}$ is selected. For Tree 1, clones marked by m_2 and m_3 are tracked since both are leaves for which a marker uniquely labeling that node is tracked. For Tree 2, the clone marked by m_2 can be tracked by $m_2 - m_3$ and the clone marked by m_3 can be tracked by m_3 alone. This yields an expected fraction 0.5 of total clonal density tracked across the tree density, which would be an optimal marker selection for this sample input.

from tissue samples, including multiregional samples, yet none to our knowledge are designed specifically for including high noise-to-signal ratio liquid biopsy samples or have been tested on comparable data. We compared results using PhyloWGS (Deshwar et al., 2015), chosen because prior experience has shown it to be particularly effective under typical cases of small numbers of samples, and an SNV-only version of our method TUSV-ext (Fu et al., 2022), which we refer to as `deconv`, chosen for its so-far unique ability to make use of SNV, SV, and CNA markers. We also applied two methods for measuring phylogenetic distance specialized to tumor phylogenies, CASet and DISC (Dinardo et al., 2020), for tree distance evaluation. For the present purposes, we assumed an initial liquid biopsy sample was sequenced along with one or more tissue samples. PhyloWGS yields good results even with only a single tissue sample, although accuracy is better the more tissue samples that are included (Fig. S2). `deconv` performs poorly by the CASet measure, although comparably to PhyloWGS by the DISC measure. These results show that there is substantial room for improvement in initial tree inference, supporting the value of refining the initial tissue tree model using subsequent ctDNA samples. They also support our decision to use PhyloWGS for real data analysis in the present work, given the limited numbers of tissue samples on which to base the initial phylogeny inference.

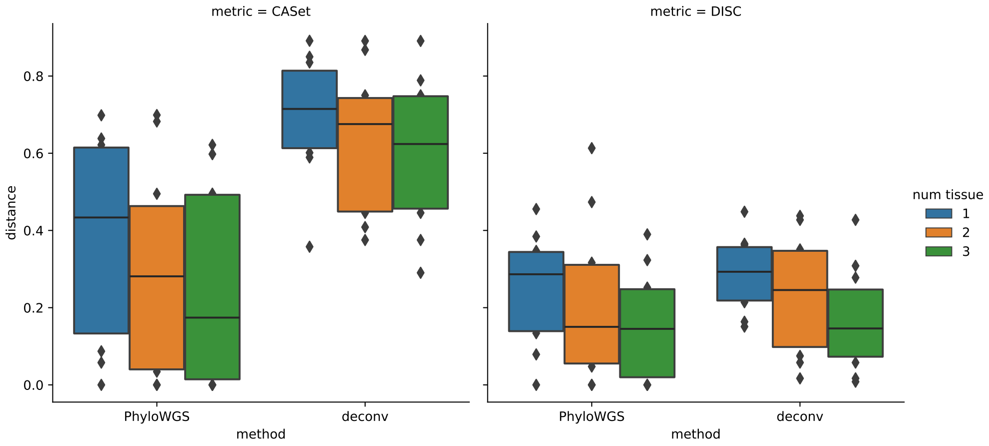

Fig. S2: Assessment of accuracy of tree inference using paired liquid biopsy and multi-regional tissue samples. The figure shows distance between true and inferred trees as function of number of samples for the PhyloWGS and deconv methods. (a) CASet distance (Dinardo et al., 2020). (b) DISC distance (Dinardo et al., 2020).
